# Annealing of gold nanostructures sputtered on polytetrafluoroethylene

**DOI:** 10.1186/1556-276X-6-588

**Published:** 2011-11-11

**Authors:** Jakub Siegel, Robert Krajcar, Zdeňka Kolská, Vladimír Hnatowicz, Václav Švorčík

**Affiliations:** 1 Department of Solid State Engineering, Institute of Chemical Technology, Technicka 5, 166 28 Prague, Czech Republic; 2 Department of Chemistry, J.E. Purkyně University, Ceské mládeze 8, 400 96 Usti nad Labem, Czech Republic; 3 Nuclear Physics Institute, Academy of Sciences of the Czech Republic, Rez, Czech Republic

## Abstract

Gold nanolayers sputtered on polytetrafluoroethylene (PTFE) surface and their changes induced by post-deposition annealing at 100°C to 300°C are studied. Changes in surface morphology and roughness are examined by atomic force microscopy, electrical sheet resistance by two point technique, zeta potential by electrokinetic analysis and chemical composition by X-ray photoelectron spectroscopy (XPS) in dependence on the gold layer thickness. Transition from discontinuous to continuous gold coverage takes place at the layer thicknesses 10 to 15 nm and this threshold remains practically unchanged after the annealing at the temperatures below 200°C. The annealing at 300°C, however, leads to significant rearrangement of the gold layer and the transition threshold increases to 70 nm. Significant carbon contamination and the presence of oxidized structures on gold-coated samples are observed in XPS spectra. Gold coating leads to a decrease in the sample surface roughness. Annealing at 300°C of pristine PTFE and gold-coated PTFE results in significant increase of the sample surface roughness.

## Introduction

Up to now, many efforts have been spent to produce smart materials with extraordinary properties usable in broad range of technological applications. In the last two decades, it has been demonstrated that properties of new prospective materials depend not only on their chemical composition but also on the dimensions of their building blocks which may consist of common materials [1,2]. Besides other interesting properties of nanostructured gold systems, such as catalytic effects or magnetism [2,3], which both originate from surface and quantum size effects, they are also extremely usable, those which are closely connected with the average number of atoms in the nanoparticles. The properties and behavior of extremely small gold particles completely differ from those of bulk materials, e.g., their melting point [2,4,5], density [6], lattice parameter [6-8], and electrical or optical properties [6,7,9] are dramatically changed. Exceptional properties of gold nanoparticles offer completely new spectrum of applications. For example, the ability to control the size and shape of the particles and their surface conjugation with antibodies allows for both selective imaging and photothermal killing of cancer cells [10-12] due to their excellent biocompatibility [13] and unique properties in surface plasma resonance [14]. Besides the medicinal applications, gold nanolayers and nanoparticles are nowadays also used in sensor technology [15] or surface-enhanced Raman spectroscopy [16].

Recently, new technique has been proposed for modification of Au nanolayer deposited on glass substrate, based on intensive post-deposition annealing [7,9]. Resulting structures are "hummock-like" isolated gold islands uniformly distributed over the substrate. The formation of new structures may be due to the accelerate diffusion and stress relaxation in gold nanolayer.

In this work, we studied the changes in surface morphology and other physico-chemical properties of gold nanolayers, sputtered on polytetrafluoroethylene surface induced by post-deposition annealing.

## Experimental details

### Substrate and Au deposition

The present experiments were performed on poly(tetrafluoroethylene) foil (PTFE, thickness of 50 956;m, *T*_g _= 126°C, and *T*_f _= 327°C) supplied by Goodfellow Ltd., UK. The gold layers were sputtered on polymer foil (2 cm in diameter). The sputtering was accomplished on Balzers SCD 050 device from gold target (supplied by Goodfellow Ltd., Huntingdon, England, UK). The deposition conditions were: DC Ar plasma, gas purity 99.995%, discharge power of 7.5 W, sputtering time 0 to 550 s. Under these experimental conditions, homogeneous distribution of gold over the substrate surface is expected [17]. Post-deposition annealing of Au-covered PTFE was carried out in air at 300°C (± 3°C) for 1 h using a thermostat Binder oven. The heating rate was 5°C.min^−1 ^and the annealed samples were left to cool in air to room temperature (RT).

### Diagnostic techniques

Electrokinetic analysis (determination of zeta potential) of pristine and Au-coated PTFE foils was accomplished on SurPASS Instrument (Anton Paar, Graz, Austria). Samples were studied inside the adjustable gap cell in contact with the electrolyte (0.001 mol.dm^−3 ^KCl). For each measurement a pair of polymer foils with the same top layer was fixed on two sample holders (with a cross-section of 20 × 10 mm^2 ^and gap between that is 100 956;m) [18]. All samples were measured four times at constant pH value with the relative error of 10%. The used method was streaming current and zeta potential was calculated by Helmholtz-Smoluchowski equation [19].

An Omicron Nanotechnology ESCAProbeP spectrometer was used to measure X-ray photoelectron spectroscopy (XPS) spectra [20]. The analyzed areas had dimensions of 2 × 3 mm^2^. The X-ray source provided monochromatic radiation of 1,486.7 eV. The spectra were measured stepwise with a step in the binding energy of 0.05 eV at each of the six different sample positions. The spectra evaluation was carried out by using CasaXPS software. The composition of the various elements is given in atomic percent disregarding hydrogen, which cannot be assessed by XPS.

Surface morphology of as-sputtered and annealed gold layers deposited for different sputtering times was examined using atomic force microscopy (AFM). The AFM images were taken under ambient conditions on a Digital Instruments CP II set-up working in tapping mode in order to eliminate damage of the sample surface. A Veeco phosphorous-doped silicon probe RTESPA-CP (Veeco, Mannheim, Germany) with spring constant of 20 to 80 N.m^−1 ^was chosen. AFM working in contact mode was also used to determine thickness of sputtered gold by scratch method. The scratch on glass substrate was made in ten different positions on as-sputtered samples and scanned in contact mode [20]. In this case, a Veeco phosphorous-doped silicon probe CONT20A-CP with spring constant 0.9 N.m^−1 ^was chosen. Thickness variations do not exceed 5%. All AFM scans were acquired at scanning rate of 1 Hz. Due to the morphology changes evoked by the annealing, the sputtered layer thickness could only be satisfactorily determined in the case of as-sputtered samples. Thus, in the case of annealed samples, the effective thickness is defined as the thickness of as-sputtered gold which is considered to be the same for annealed structures deposited for the corresponding deposition time.

Sheet resistance (*R*_s_) of the gold layers was measured by standard two point method. Two gold contacts, defining measured area (about 50 nm thick) on the layer surface were prepared by sputtering. We define an electrically continuous layer as a layer, where the declining sheet resistance reaches a saturated minimum.

## Results and discussion

**Figure 1:**
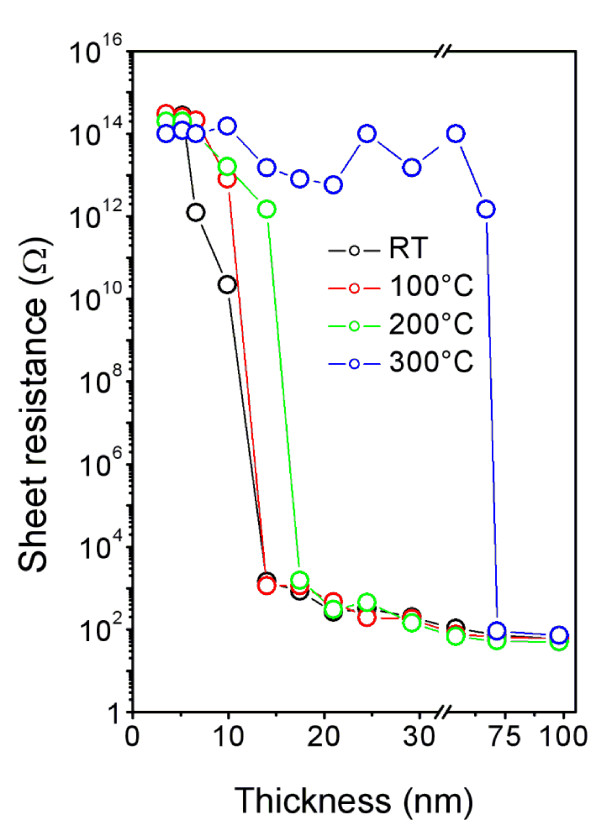
**Sheet resistance**. Dependence of the sheet resistance (*R*_s_) on Au layer thickness for as-sputtered samples (RT) and the samples annealed at 100°C, 200°C, 300°C.

The dependence of the electrical sheet resistance (*R*_s_) of the gold layer on its thickness before and after annealing (at 100°C, 200°C, and 300°C) is shown in Figure 1. For the as-sputtered samples the sheet resistance decreases rapidly in the narrow thickness from 10 to 15 nm when an electrical continuous gold coverage is formed. The resulting sheet resistance is saturated at a level of ca 200 Ω. From the measured *R_s _*and effective layer thickness, layer resistivity *R *(ohm centimeter) was calculated, which appears to be orders of magnitude higher than that reported for metallic bulk gold (*R*_Au_^bulk ^= 2.5 × 10^−6 ^Ω cm [21], e.g., for 100-nm thick Au layer *R*_Au_^100 nm ^= 1 × 10^−3 ^Ω cm). The higher resistivity of thin gold structures is due to the size effect in accord with the Matthiessen rule [22]. It is an empirical rule, which states that the total resistivity of a crystalline metallic specimen is the sum of the resistivity due to thermal agitation of the metal ions of the lattice and the resistivity due to the presence of imperfections in the crystal. Imperfections such as impurity atoms, interstitials, dislocations, and grain boundaries scatter conduction electrons because in their immediate vicinity, the electrostatic potential differs from that of the perfect crystal. Owing to the limited material dimensions and resulting high surface-to-bulk ratio, the development of new allowed surface states occurs which affects local electrostatic potential. As a result, conduction electrons are scattered during electrical measurements performed on thin metal layers which causes higher resistivity compared to bulk material. Annealing at temperatures below 200°C causes only mild shift in the resistance curve towards thicker layers. Transition from electrically discontinuous to electrically continuous layer in case of low temperature annealed samples is more gradual and occurs between the effective layer thicknesses from 10 to 20 nm regarding the annealing temperature. After annealing at 300°C a dramatic change in the resistance curve is observed. The annealed layers are electrically discontinuous up to the Au effective thickness of 70 nm above which the continuous coverage is created and a percolation limit is overcome. However, for longer sputtering times up to 550 s, the sheet resistance changes slowly and it achieves a saturation which is observed on the as-sputtered layers and layers annealed at low temperatures.

Besides of the sheet resistance, measurement information on the layer structure and homogeneity can be obtained in another way too. Here, complementary information on the layer homogeneity is obtained from XPS spectra.

**Figure 2:**
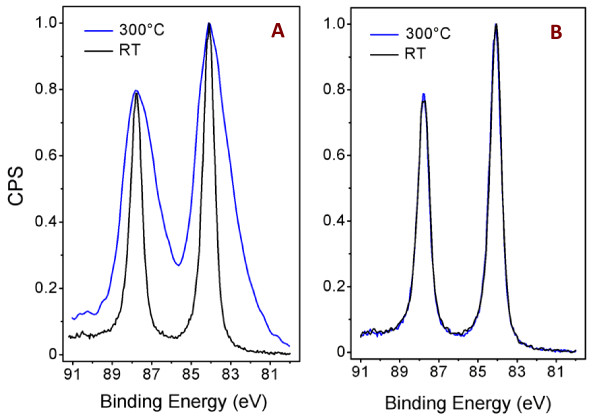
**Normalized XPS spectra**. Intensity normalized XPS spectra (line Au (4f)) of 20-nm **(A) **and 80-nm **(B) **thick sputtered Au layers on PTFE before (black line) and after (blue line) annealing at 300°C.

Figure 2A,B shows intensity normalized XPS spectra (line Au 4f) of 20- and 80-nm thick sputtered gold layers, respectively. Black line refers to as-sputtered layer and blue line to one annealed at 300°C. Annealing of the 80-nm thick gold layer does not change the XPS spectrum. In contrast, the annealing of the 20-nm thick layer results in strong broadening of both lines which is due to the sample charging in the course of the XPS analysis. The charging is closely related to the change in the layer morphology: from electrically continuous one for as-sputtered sample to discontinuous one after the annealing procedure [9]. This observation is in agreement with above described results of the sheet resistance measurements (see Figure 1).

Concentrations of chemical elements on the very sample surface (accessible depth of 6 to 8 atomic layers) determined from XPS spectra are summarized in Table 1. The XPS data were obtained for the samples with 20- and 80-nm thick gold layers, both as-sputtered and annealed at 300°C. Total carbon concentration and the carbon concentration coming from PTFE (calculated from XPS data) are shown in columns 1 and 2 of the table, respectively. Major part of the carbon is due to sample contamination. Fluorine to PTFE carbon ratio F/C^PTFE ^is close to that expected for PTFE (about 2). By the annealing at 300°C, the ratio decreases to 1.7 for both layer thicknesses. The decrease may be due to reorientation of polar C-F groups induced by thermal treatment. Oxygen detected in the samples may result from oxygen incorporation during gold sputtering which may be accompanied by partial degradation and oxidation of PTFE macromolecular chain or degradation products. Subsequent annealing leads to reorientation of the oxidized groups toward the sample bulk and corresponding decrease of the surface concentration of oxygen. The same effects have been observed earlier on plasma-modified polyolefines [23]. It is also evident from Table 1 that annealing causes resorption of contamination carbon both hydrogenated and oxidized one [24]. Changes in the morphology of the gold layer after the annealing are manifested in changes of the gold and fluorine concentrations as observed in XPS spectra. After the annealing, the observed gold concentration decreases and fluorine concentration increases dramatically, these changes clearly indicate formation of isolated Au islands similarly as in case of Au-coated glass substrate [9].

**Table 1:** Atomic concentrations

AU layer thickness	Temperature	Atomic concentrations of elements in at.%					
		C	C^PTFE^	O	Au	F	F/C^PTF^
20 nm	RT	43.5	4.4	6.5	41.6	8.5	1.93
	300°C	37.8	34.8	0.4	3.4	58.4	1.68
80 nm	RT	41.0	3.1	4.4	48.6	6.0	1.94
	300°C	36.8	27.2	1.2	14.8	47.2	1.74

Atomic concentrations (in atomic percent) of C (1s), O (1s), Au (4f), and F(1s) in Au-sputtered PTFE samples with Au effective thickness 20 and 80 nm after deposition (RT) a after annealing (300°C) measured by XPS. C^PTFE^, calculated concentration from XPS data of carbon (in atomic percent) originating from PTFE only; F/C^PTFE^, stands for fluorine to PTFE carbon ratio.

**Figure 3:**
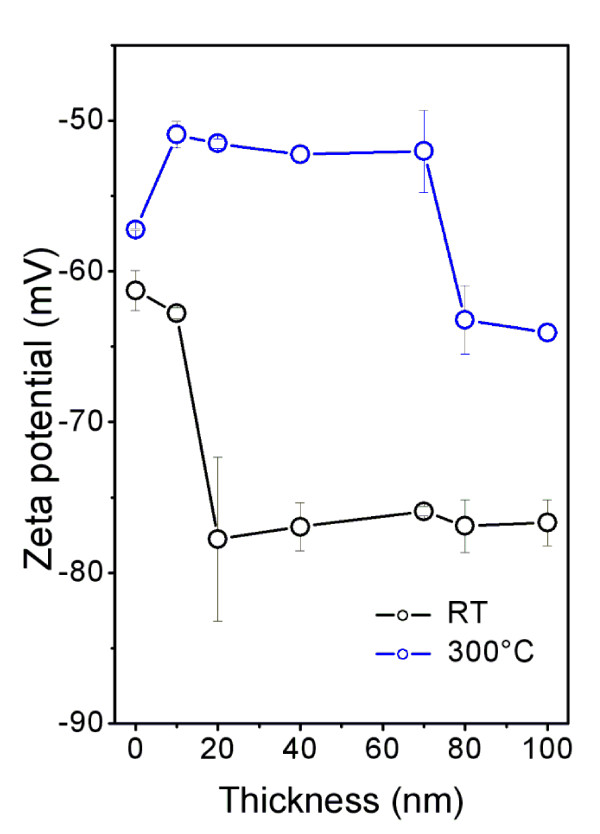
**Zeta potential**. Dependence of zeta potential on the Au layer thickness for as-sputtered samples (RT) and the samples annealed at 300°C.

**Figure 4:**
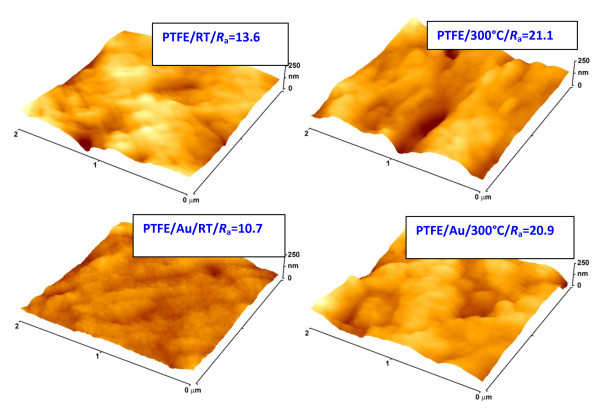
**AFM images of pristine (PTFE) and Au-coated (PTFE/Au) samples (thickness of 20 nm)**. Before (RT) and after annealing at 300°C. Numbers in frames are measured surface roughnesses *R*_a _in nanometers.

Another quantity characterizing the structure of the sputtered gold layers is zeta potential determined from electrokinetic analysis. Dependence of zeta potential on the gold layer thickness for as-sputtered samples (RT) and annealed samples at 300°C is shown in Figure 3. For as-sputtered samples and very thin gold layers, the zeta potential is close to that of pristine PTFE due to the discontinuous gold coverage since the PTFE surface plays dominant role in zeta potential value. Then, for thicker layers, where the gold coverage prevails over the original substrate surface, the zeta potential decreases rapidly and for the thicknesses above 20 nm remains nearly unchanged, indicating total coverage of original substrate by gold. For annealed samples, the dependence on the layer thickness is quite different. It is seen that the annealing leads to a significant increase of the zeta potential for very thin layers. This increase may be due to thermal degradation of the PTFE accompanied by production of excessive polar groups on the polymer surface, which plays the important role when the gold coverage is discontinuous. Moreover, the surface roughness increases at this moment too (see Table 1 and Figure 4 below) [25]. Then, for medium thicknesses, ranging from 20 to 70 nm, the zeta potential remains unchanged and finally it decreases again for higher thicknesses due to the formation of continuous gold coverage. It appears that the results of electrokinetic analysis (Figure 3) and measurement of the sheet resistance (Figure 1) are highly correlated. The rapid decrease in the sheet resistance occurs at the same layer thickness as the decrease in zeta potential. Both correlated changes are connected with creation of continuous, conductive gold coverage. Another interesting fact is that even for the layers with thicknesses above 80 nm, the values of the zeta potential measured on as-sputtered and annealed samples differ significantly. This can be due to higher fluorine concentration in the annealed samples and the fact that the C-F bond is more polar and exhibits higher wettability. It should be also noted that the value of the zeta potential may be affected by the surface roughness too (see below). In general, it follows that the thicker the gold coverage the lower the zeta potential is, reflecting the electrokinetic potencial of metal itself.

The changes in the surface morphology after the annealing were studied by AFM. AFM scans of pristine and Au-coated (20 nm) samples before and after annealing are presented in Figure 4. One can see that the annealing causes a dramatic increase in the surface roughness of the pristine polymer. Since the annealing temperature markedly exceeds PTFE glassy transformation temperature (*T*_g_^PTFE ^= 126°C) the increase in the surface roughness is probably due to thermally induced changes of PTFE amorphous phase. The gold sputtering leads to a measurable reduction of the sample surface roughness. The reduction may be due to preferential gold growth in hollows at the PTFE surface. Annealing of the gold-coated sample leads to significant increase of the surface roughness too. In this case, the increase is a result of both, the changes in the surface morphology of underlying PTFE and the changes in the morphology of the gold layer. After annealing, the surface roughness of pristine and gold-coated samples is practically the same. This finding is in contradiction with similar study accomplished on gold layers deposited on glass substrate [9]. Possible explanation of this fact probably lies in much better flatness of the glass substrate and in lower thermal stability of PTFE substrate during annealing.

## Conclusions

The properties of thin gold layers sputtered on the PTFE substrate and their changes after annealing at 100°C to 300°C were studied by different methods. Chemical composition, electrical conductivity, surface morphology, and zeta potential of the layers as a function of the layer thickness were determined. Attention was focused on the transition from partial to complete gold coverage of PTFE substrate. From the measurement of the sheet resistance the transition from discontinuous to continuous gold coverage was found at the layer thicknesses 10 to 15 nm for as-sputtered samples. After annealing at 300°C, the transition point increase to about 70 nm, the increase indicating substantial rearrangement of the gold layer. The rearrangement is confirmed also by XPS measurement and an electrokinetic analysis. By XPS measurement, contamination of the gold coated PTFE samples with carbon and the presence of oxidized structures created during gold sputtering were proved. The annealing results in significant increase of the surface roughness of both pristine- and gold-sputtered PTFE.

## Competing interests

The authors declare that they have no competing interests.

## Authors' contributions

JS participated in surface morphology measurements and designed and drafted the study. RK carried out resistance measurements together with its evaluation. ZK carried out zeta potential measurements. VH and VŠ conceived of the study and participated in its coordination. All authors read and approved the final manuscript.
